# Supplementary material for: Predicting the Development of Anti-Drug Antibodies against Recombinant alpha-Galactosidase A in Male Patients with Classical Fabry Disease
Source: Int J Mol Sci. 2020 Aug 12;21(16):5784. doi: 10.3390/ijms21165784 (PMC7460974; doi:10.3390/ijms21165784)
Supplement: Supplementary file 1 [file ijms-21-05784-s001.pdf]

iADA prediction script

Sanne Jolien van der Veen

June 26th 2020

Chapters

1. Load required packages
2. check data type and inspect missing data
3. Data imputation using chained equations (mice)
4. Distribution of variables
5. Load additional packages
6. Regular logistic regression model
7. Checking assumptions
8. Logistic regression model(repeated CV)
9. Random forest model (repeated CV)
10. Post hoc analyses (high risk missense mutation?)
11. External cohort

```
# Text in orange is added to explain the rationale behind each step and/or gi
ve additional information.
```

1. load required packages

```
library('mice')
library(readxl)
library(ggplot2)
```

2. Check data type and inspect missing data

```
summary(dataset)

##      random     ADA      site          DNAmut     dnalocation
##  1       :  1   0:62   1:39   c.1025G>A: 9   Min.   :   1.0
##  2       :  1   1:58   2:50   c.679C>T : 8   1st Qu.: 407.5
##  3       :  1          3:31   c.406G>T : 4   Median : 717.0
##  4       :  1                 c.658C>T : 4   Mean   : 712.1
##  5       :  1                 c.901C>T : 4   3rd Qu.:1025.0
##  6       :  1                 IVS3+1G>A: 4   Max.   :1246.0
##  (Other):114                 (Other)  :87   NA's   :8
##     mutationtype  ageertstart       lysogb3          ADATiter
##  duplication: 1   Min.   : 9.55   Min.   : 38.10   Min.   :    0.00
##  frameshift :17   1st Qu.:26.69   1st Qu.: 84.05   1st Qu.:    2.00
```

```
##  missense    :58   Median :36.56   Median :113.30   Median :    4.33
##  nonsense    :33   Mean   :35.64   Mean   :109.37   Mean   :  576.61
##  other       : 1   3rd Qu.:43.36   3rd Qu.:127.10   3rd Qu.:  107.48
##  splice site:10   Max.   :63.47   Max.   :177.80   Max.   :32645.49
##                                    NA's   :33
##  firsttreatmenttype firstdose
##  Fabrazyme:75       0.2:51
##  Replagal :45       0.5: 4
##                     1  :65
##
```

```r
# Add frameshift mutations to the group of nonsensemutations and add duplicat
ions and splice site mutations to the 'other' mutations.

dataset$mutationtype[dataset$mutationtype == "frameshift"] <- "nonsense"
dataset$mutationtype[dataset$mutationtype == "duplication"] <- "other"
dataset$mutationtype[dataset$mutationtype == "splice site"] <- "other"


# Inspect number of missing variables. (N.b. mutation location is missing whe
n the mutation is not in the coding aGalA sequence)

sapply(dataset, function(x) sum(is.na(x)))
```

```
##             random                ADA               site
##                  0                  0                  0
##             DNAmut        dnalocation       mutationtype
##                  0                  8                  0
##        ageertstart            lysogb3           ADATiter
##                  0                 33                  0
## firsttreatmenttype          firstdose
##                  0                  0
```

```r
# Inspect number of unique entries in each group.

sapply(dataset, function(x) length(unique(x)))
```

```
##             random                ADA               site
##                120                  2                  3
##             DNAmut        dnalocation       mutationtype
##                 71                 68                  3
##        ageertstart            lysogb3           ADATiter
##                119                 86                 70
## firsttreatmenttype          firstdose
##                  2                  3
```

```r
# calculate what percentage of total data is missing


sum(is.na(dataset))/prod(dim(dataset))
```

```
## [1] 0.03106061
```

3. Data imputation using chained equations

```
# Mice is used to impute missing values (DNA location (N=8) and baseline lyso
Gb3(N=33)
```

```
set.seed(42)
initial = mice(dataset, maxit=5)
predM = initial$predictorMatrix


# We exclude the following from use in imputation: explanation is given under
neath the code.


predM[, c("random")]=0
# "random" represents the patientnr (1-120)and has no influence on any values
predM[, c("site")]=0
# "site" represents the hospital at which the patient is treated and has no i
nfluence on any values
predM[, c("DNAmut")]=0
# exact mutation could not be used as there are 71 unique values in 120 patie
nts. mutationtype is used
predM[, c("firsttreatmenttype")]=0
predM[, c("firstdose")]=0
# as treatment is not related to baseline characteristics

set.seed(42)
# Make 5 different datasets (To test the consistency of imputation, 5 differe
nt datasets with imputed values are generated and compared)


imputed=mice(dataset, predictorMatrix=predM, m=5)
```

# Show distribution of imputed values in each of the 5 imputed datasets. Blue dots represent real values. Red dots are the imputed values. X axis 1-5 are the different created datasets. Distribution of imputation is considered consistant.

```
stripplot(imputed, pch = 20, cex = 1.2)
```

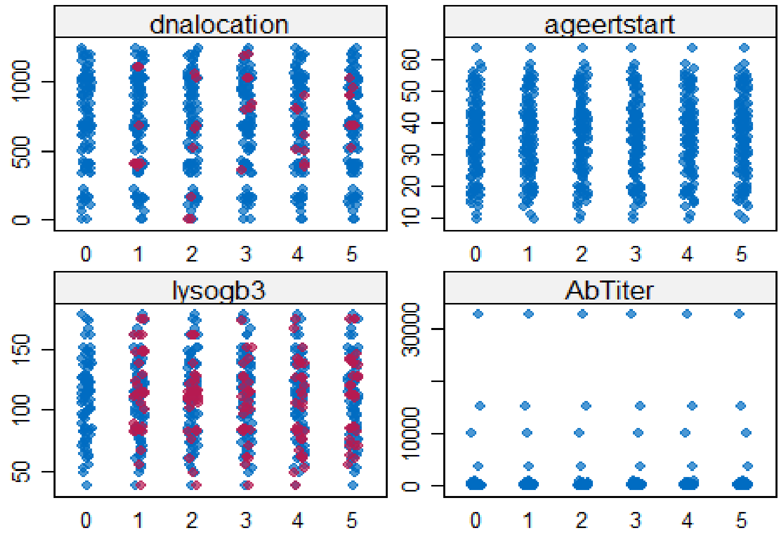


```
# Visualize the differences in mean and SD in all imputed datasets for each iteration
```

```
plot(imputed)
```

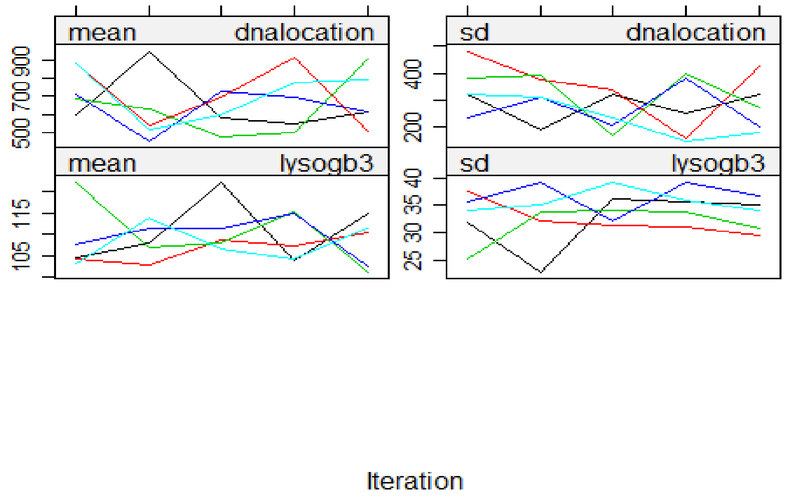


```
# Complete and aggregate datasets: The mean of all 5 individual imputations is used as the final imputed value.
```

```
imputedall <- mice::complete(imputed, action="long")
aggregated <- setNames(aggregate(list(imputedall$lysogb3, imputedall$dnalocation), by=list(imputedall$random), FUN=mean),c("random", "lysoimputed", "dnalocimputed"))
imputedclean <- merge(prediction[, -c(5,8)], aggregated, by=c("random"))
```

4. Distribution of variables

```r
# Imputed dataset is split in 2 (iADA positive and iADA negative to check baseline characteristics)

ADAplus=imputedclean[imputedclean$ADA==1,]
ADAmin=imputedclean[imputedclean$ADA==0,]

# Summary of variables in ADA positive group

summary(ADAplus)

##      random    ADA    site          DNAmut        mutationtype
##  1      : 1   0: 0   1:23   c.1025G>A      : 6   duplication: 0
##  2      : 1   1:58   2:24   c.658C>T       : 4   frameshift : 0
##  3      : 1          3:11   c.679C>T       : 4   missense   :21
##  4      : 1                 c.1025G>T      : 2   nonsense   :33
##  6      : 1                 c.157_160delAACC: 2   other      : 4
##  7      : 1                 c.677G>A       : 2   splice site: 0
##  (Other):52                 (Other)        :38
##   ageertstart       ADATiter       firsttreatmenttype firstdose
##  Min.   : 9.55   Min.   :    7.00   Fabrazyme:44       0.2:18
##  1st Qu.:29.30   1st Qu.:   22.42   Replagal :14       0.5: 2
##  Median :37.83   Median :  112.50                      1  :38
##  Mean   :36.48   Mean   : 1190.92
##  3rd Qu.:43.11   3rd Qu.:  260.00
##  Max.   :58.46   Max.   :32645.49
##
##   lysoimputed    dnalocimputed
##  Min.   : 38.1   Min.   :   1.0
##  1st Qu.:108.2   1st Qu.: 658.0
##  Median :123.0   Median : 752.5
##  Mean   :121.5   Mean   : 732.7
##  3rd Qu.:137.9   3rd Qu.:1025.0
##  Max.   :177.8   Max.   :1246.0
##

# Summary of variables in ADA positive group

summary(ADAmin)

##      random    ADA    site            DNAmut        mutationtype
##  5      : 1   0:62   1:16   c.406G>T         : 4   duplication: 0
##  10     : 1   1: 0   2:26   c.679C>T         : 4   frameshift : 0
##  12     : 1          3:20   c.1025G>A        : 3   missense   :37
##  13     : 1                 IVS3+1G>A        : 3   nonsense   :17
##  14     : 1                 c.1081G>A        : 2   other      : 8
##  15     : 1                 c.1208-1211delTAA: 2   splice site: 0
##  (Other):56                 (Other)          :44
```

```
##   ageertstart      ADATiter      firsttreatmenttype firstdose
##  Min.   :13.39   Min.   :0.000   Fabrazyme:31       0.2:33   
##  1st Qu.:24.31   1st Qu.:0.000   Replagal :31       0.5: 2   
##  Median :35.10   Median :2.000                      1  :27   
##  Mean   :34.85   Mean   :1.938                               
##  3rd Qu.:44.22   3rd Qu.:3.000                               
##  Max.   :63.47   Max.   :5.000                               
##                                                              
##   lysoimputed      dnalocimputed   
##  Min.   : 48.30   Min.   :  53.0  
##  1st Qu.: 82.00   1st Qu.: 406.5  
##  Median : 96.15   Median : 679.0  
##  Mean   : 97.37   Mean   : 689.9  
##  3rd Qu.:114.85   3rd Qu.: 959.0  
##  Max.   :148.60   Max.   :1221.0  
## 

# Visualization of variables associated with increased iADA risk. Code is hid
den. Figures are edited in adobe illustrate for final figures.
```

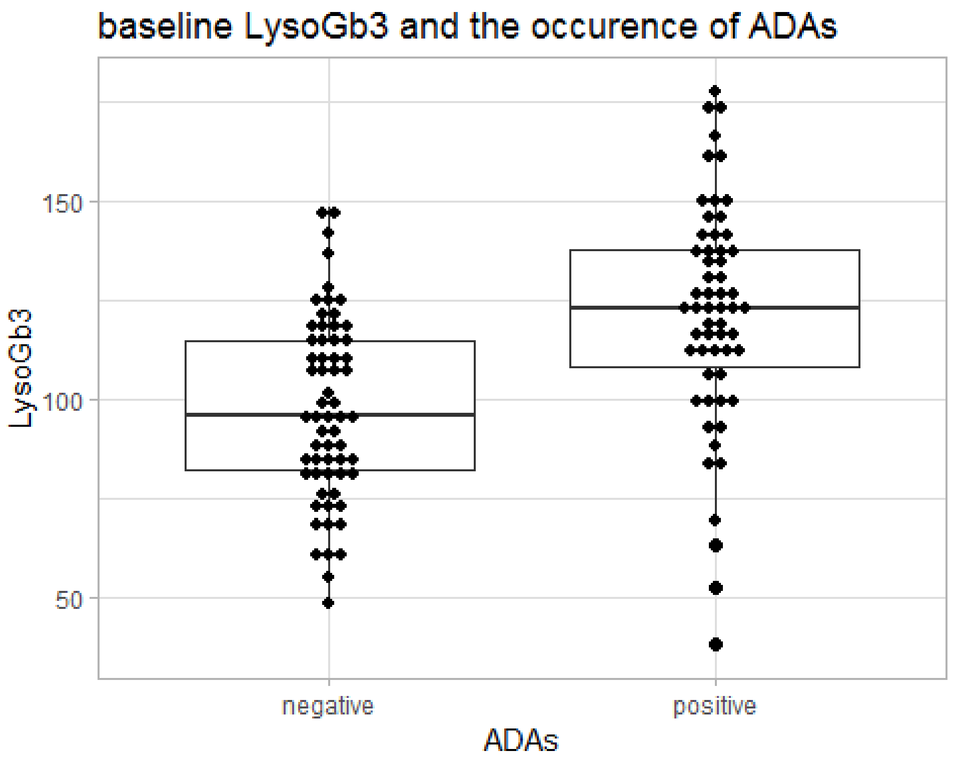

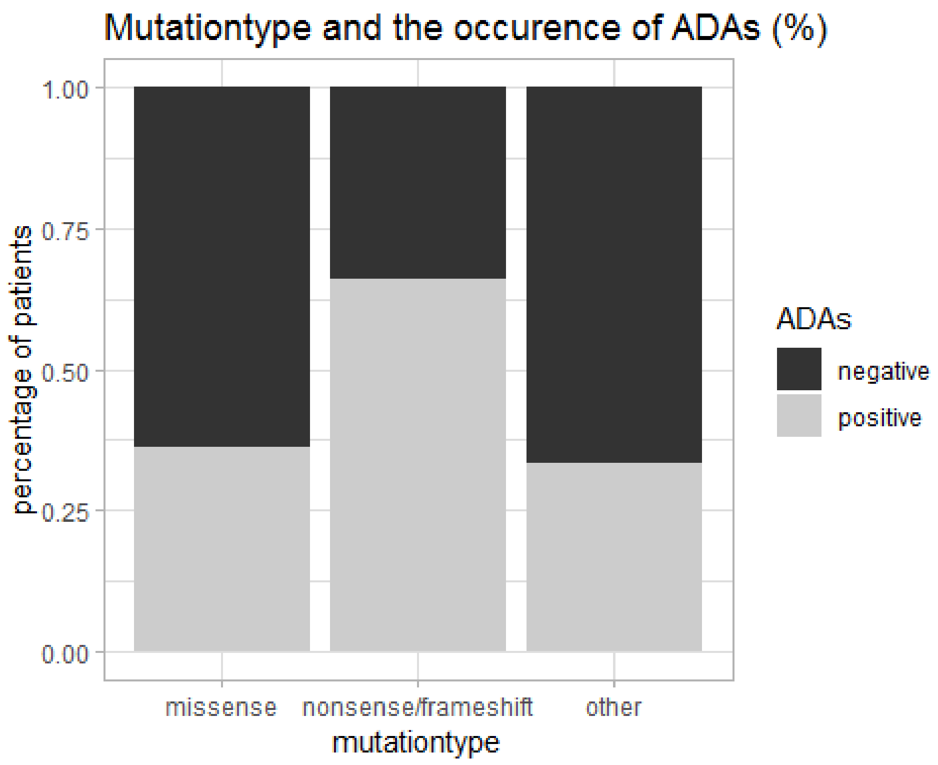


```
#As there is no difference in the occurence of iADAs between missense and 'ot
her' mutations, those have been joined into one group to reduce variables.

imputedclean$mutatietype[imputedclean$mutatietype == "missense"] <- "other"
```

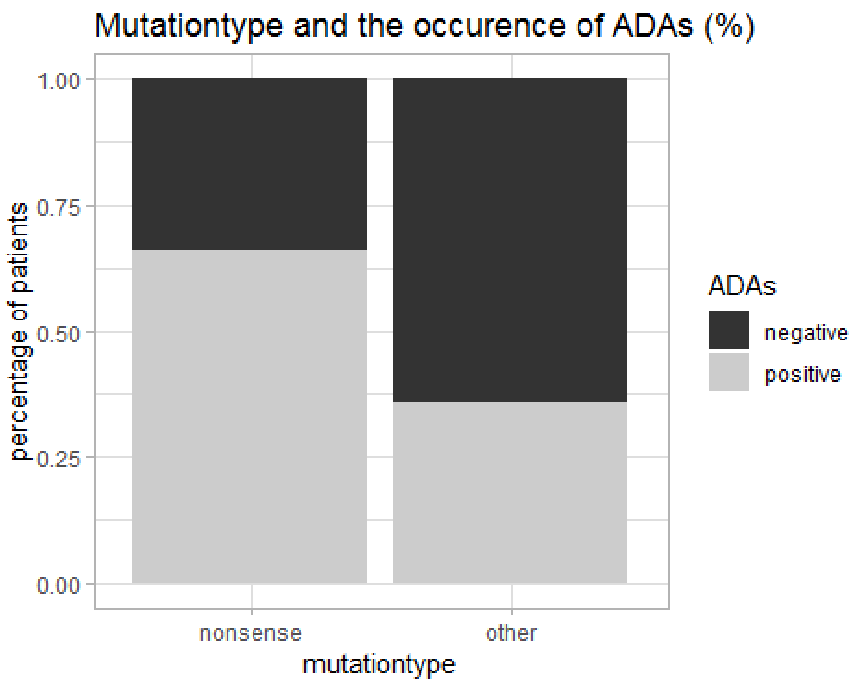

```
# First treatment type.
```

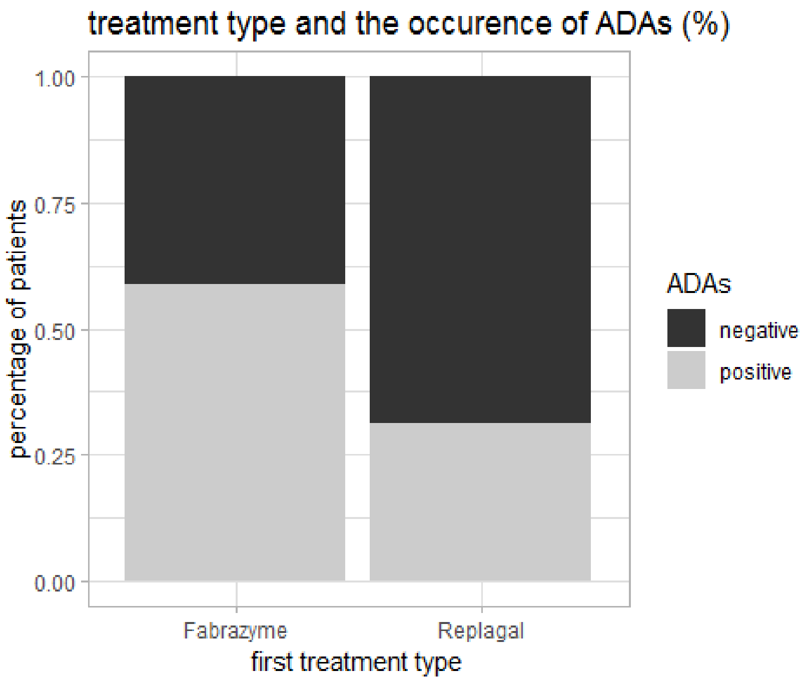


```
#first dose
```

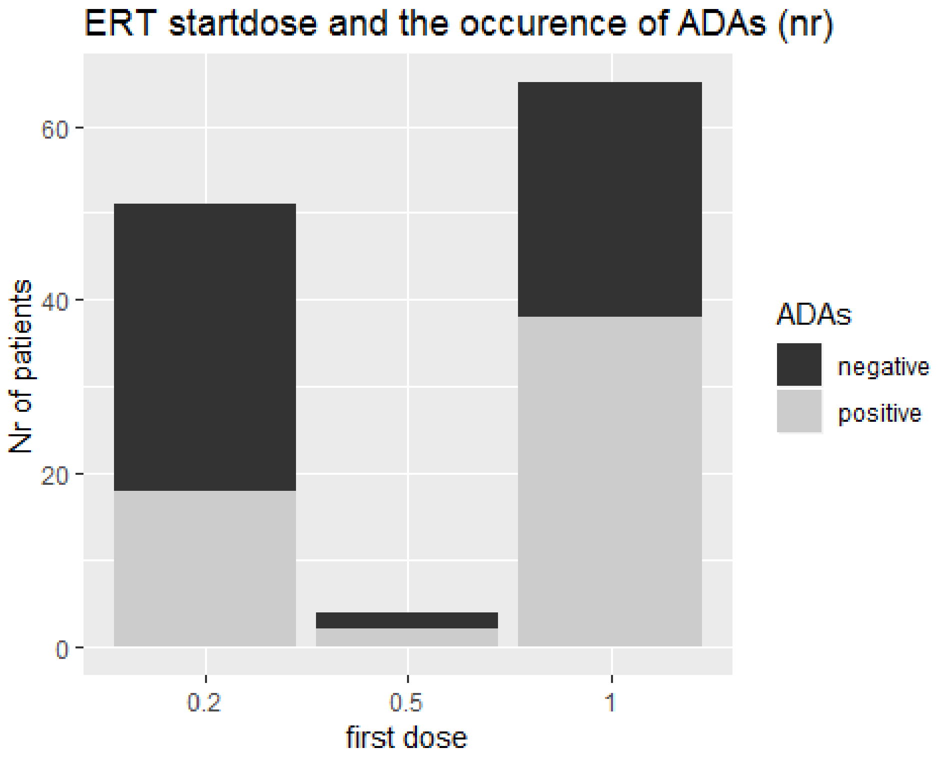

```
# As dose and treatment type are strongly linked (Replagal can only be dosed at 0.2mg/kg while Fabrazyme is recommended at 1mg/kg), both will be tested in the LR model, but only one may stay in the model as LR cannot deal with co-linearity.
```

5.load additional packages

```
library(caret)
library(randomForest)
library(pROC)
library(ROCR)
library(tidyverse)
library(car)
library(VIM)
library(corrplot)
library(BaylorEdPsych)
library(mvnmle)
library(caTools)
library(broom)
library(dplyr)
library(plotROC)

# A ‘simple’ LR model was built to test and eliminate variables by backwards selection until only variables that were significantly associated with ADA risk remained. Outcome was used to test assumptions. (only the final model is shown)
```

6. Regular logistic regression model

```
fullhandmodel=glm(ADA~mutationtype+lysoimputed+firsttreatmenttype, data=imputedclean, family='binomial')
summary(fullhandmodel)

##
## Call:
## glm(formula = ADA ~ mutationtype + lysoimputed + firsttreatmenttype,
##     family = "binomial", data = imputedclean)
##
## Deviance Residuals:
##     Min       1Q   Median       3Q      Max
## -2.0356  -0.8751  -0.4152   0.9481   2.1485
##
## Coefficients:
##                            Estimate Std. Error z value Pr(>|z|)
## (Intercept)                -2.54806    1.08188  -2.355 0.018512 *
## mutationtypeother           -0.85785    0.44428  -1.931 0.053498 .
## lysoimputed                 0.03156    0.00896   3.523 0.000427 ***
## firsttreatmenttypeReplagal -1.21860    0.44566  -2.734 0.006250 **
## ---
## Signif. codes:  0 '***' 0.001 '**' 0.01 '*' 0.05 '.' 0.1 ' ' 1
```

```
##
## (Dispersion parameter for binomial family taken to be 1)
##
##     Null deviance: 166.22  on 119  degrees of freedom
## Residual deviance: 131.39  on 116  degrees of freedom
## AIC: 139.39
##
## Number of Fisher Scoring iterations: 4
```

7. Checking assumptions

#source of tutorial: (http://www.sthda.com/english/articles/36-classification-methods-essentials/148-logistic-regression-assumptions-and-diagnostics-in-r/)

7.1. Outcome is binary

Yes: iADA- and iADA+

7.2. There is a linear relationship between the logit of the outcome and prediction variables.

Yes: out of the following only lysoGb3 is added in the GLM model.

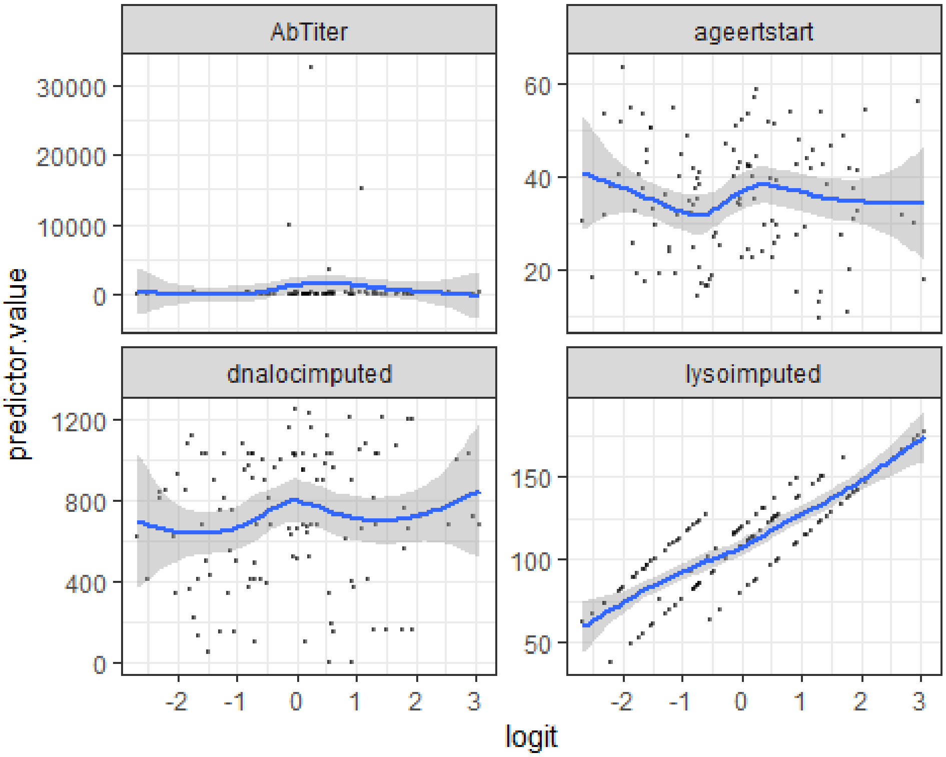

7.3. There are no influential values (extreme values or outliers in the continuous predictors)

influential factors: cooks distance

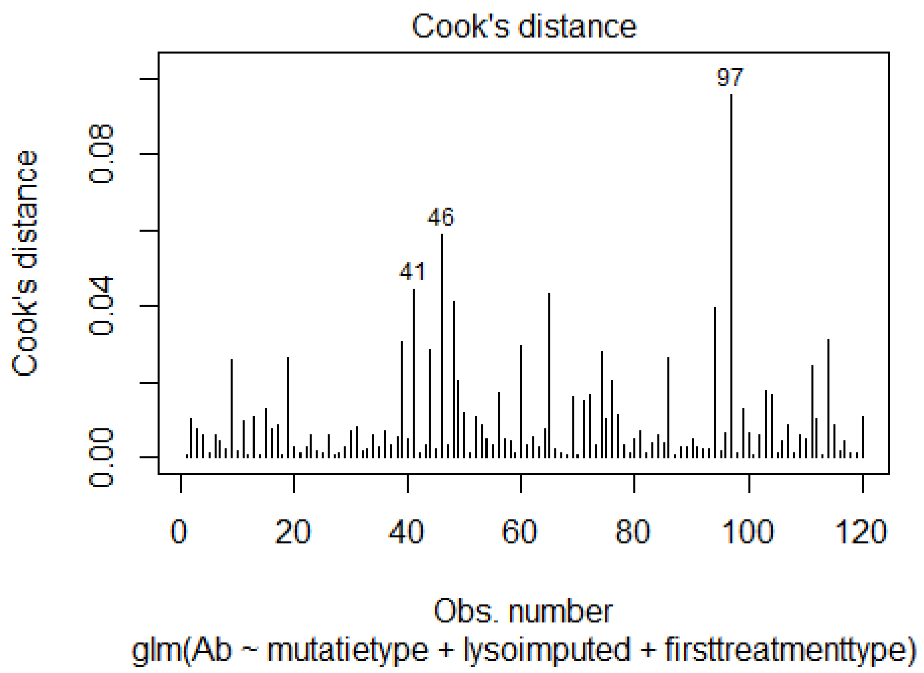


```
## # A tibble: 3 x 12
##   Ab    mutatietype lysoimputed firsttreatmentt~ .fitted .se.fit .resid
##   <fct> <fct>             <dbl> <fct>              <dbl>   <dbl>  <dbl>
## 1 0     other             149.  Fabrazyme           1.28   0.514  -1.75
## 2 1     other              52.7 Fabrazyme          -1.74   0.548   1.95
## 3 1     other              38.1 Fabrazyme          -2.20   0.660   2.15
## # ... with 5 more variables: .hat <dbl>, .sigma <dbl>, .cooksd <dbl>,
## #   .std.resid <dbl>, index <int>
```

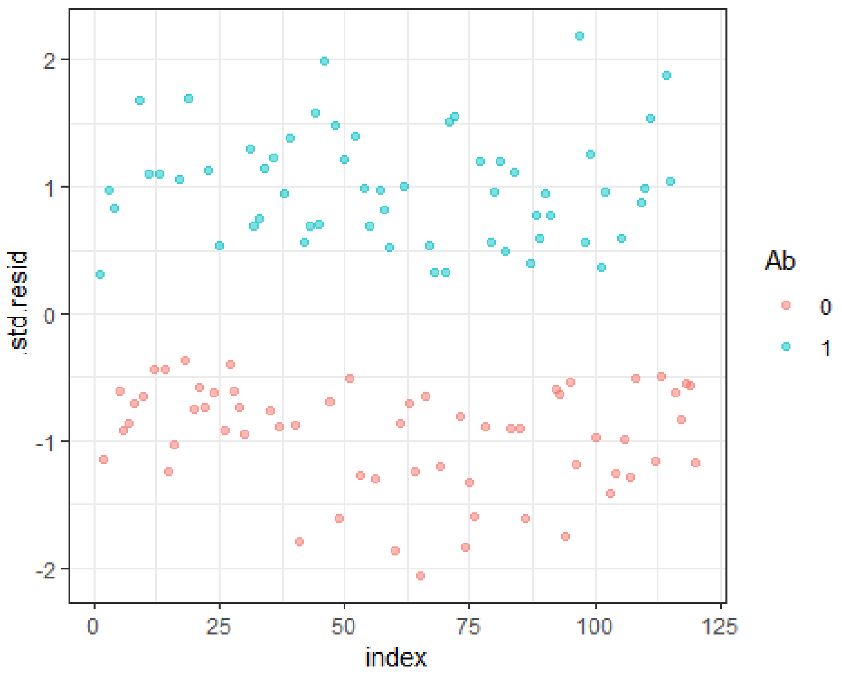

7.4.There is no high intercorrelations (i.e. multicollinearity among the predictors.)

```
##        mutatietype         lysoimputed firsttreatmenttype
##           1.062208            1.049844           1.024860
```

Advanced prediction models

8. Logistic regression model (repeated CV)

```
ADA = factor(ifelse(imputedclean$Ab == 1, "VALID", "INVALID"), levels = c("VA
LID", "INVALID"))
imputedclean[, c("ADA","random", "site", "ADATiter", "dnalocimputed","dnalocs
impel","ageertstart","firstdose", "DNAmut")] = NULL

set.seed(42)
glm_model = train(y = ADA, x = imputedclean, method = "glm", metric = "ROC",
                  trControl=trainControl(method="repeatedcv", number=10, repe
ats = 10,
                                         classProbs = TRUE, savePredictions =
TRUE,
                                         summaryFunction = twoClassSummary),
control = list(maxit = 50))
print(glm_model)

## Generalized Linear Model
##
## 120 samples
##   3 predictor
##   2 classes: 'VALID', 'INVALID'
##
```

```
## No pre-processing
## Resampling: Cross-Validated (10 fold, repeated 10 times)
## Summary of sample sizes: 109, 108, 108, 108, 107, 108, ...
## Resampling results:
##
##   ROC        Sens   Spec
##   0.7696032  0.699  0.7054762
```

```
varImp(glm_model)
```

```
## glm variable importance
##
##                             Overall
## lysoimputed                  100.00
## firsttreatmenttypeReplagal    50.47
## mutatietypeother               0.00
```

```
summary(glm_model)
```

```
##
## Call:
## NULL
##
## Deviance Residuals:
##     Min       1Q   Median       3Q      Max
## -2.1485  -0.9481   0.4152   0.8751   2.0356
##
## Coefficients:
##                            Estimate Std. Error z value Pr(>|z|)
## (Intercept)                 2.54806    1.08188   2.355 0.018512 *
## mutatietypeother            0.85785    0.44428   1.931 0.053498 .
## firsttreatmenttypeReplagal  1.21860    0.44566   2.734 0.006250 **
## lysoimputed                -0.03156    0.00896  -3.523 0.000427 ***
## ---
## Signif. codes:  0 '***' 0.001 '**' 0.01 '*' 0.05 '.' 0.1 ' ' 1
##
## (Dispersion parameter for binomial family taken to be 1)
##
##     Null deviance: 166.22  on 119  degrees of freedom
## Residual deviance: 131.39  on 116  degrees of freedom
## AIC: 139.39
##
## Number of Fisher Scoring iterations: 4
```

```
predsGLM= prediction(glm_model$pred$VALID, glm_model$pred$obs)
perfglm=performance(predsGLM, "tpr", "fpr")
plot(perfglm, main="ROC GLM model", colorize=TRUE, colorkey.relwidth=0.5,lwd=
3,
     xaxis.cex.axis=1.2, yaxis.cex.axis=1.2, cex.lab=1.2, cex.main=2)
abline(a=0,b=1)
```

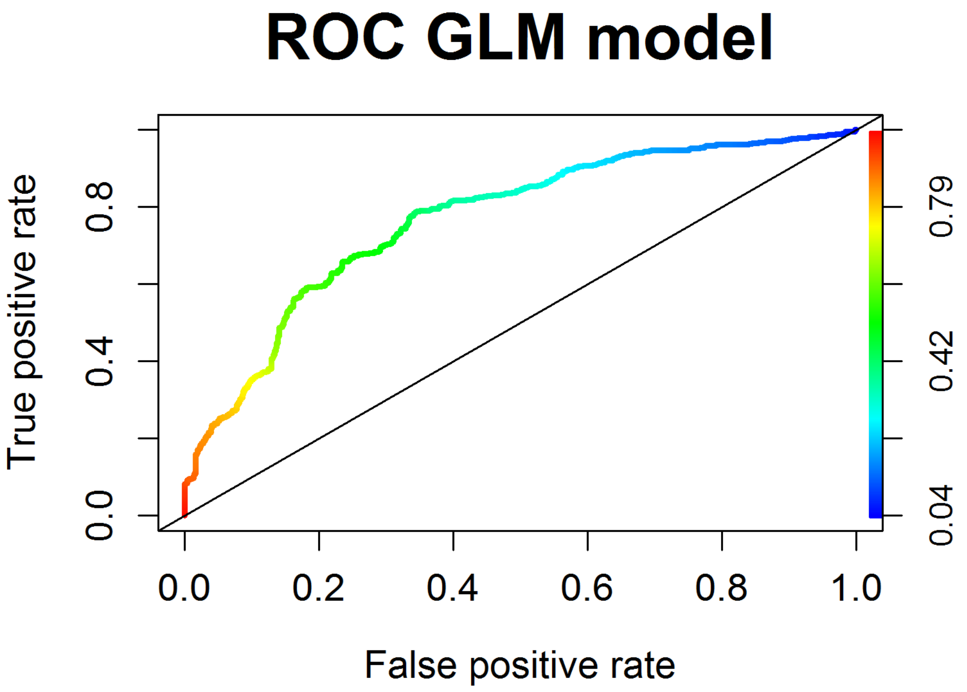


```
# Due to the 10 fold cross validation, 10 predicted outcomes are available pe
r patientl (As each patient has been in the training set 90 times and in the
validation set 10 times. The mean of 10 predicted outcomes of each patient is
used in the final outcome.


outcome=glm_model$pred
outcomemean= aggregate(outcome[,3], list(outcome$rowIndex), mean)
predsGLMmean= prediction(outcomemean$x, ADA)
perfglmmean=performance(predsGLMmean, "tpr", "fpr")

handpred=prediction(outcomemean$x, ADA)
handeval=performance(handpred, "acc")
handsens=performance(handpred, "sens")
handspec=performance(handpred, "spec")

max=which.max(slot(handeval, "y.values")[[1]])
accglm=slot(handeval, "y.values")[[1]][max]
cutglm=slot(handeval, "x.values")[[1]][max]
print(c(Accuracy=accglm, Cutoff=cutglm))

##  Accuracy    Cutoff
## 0.7250000 0.5283533

#combine outcome and predictions (mean)
merged=cbind(ADA, outcomemean)
```

```
merged$pred52=ifelse(merged$x <= 0.528, "INVALID", "VALID")
merged=merged%>%
  rename(
    patient=Group.1,
    prediction= x, observation = ADA
  )

table=table(merged$pred52, merged$observation)
table

##
##            VALID INVALID
##   INVALID    18      47
##   VALID      40      15

#sensitivity is:
40/(40+18)

## [1] 0.6896552

#specificity is:
47/(47+15)

## [1] 0.7580645

#visualisation model performance
plot(perfglmmean, main="ROC GLM model", colorize=TRUE, colorkey.relwidth=0.5,
lwd=4,
     xaxis.cex.axis=1.2, yaxis.cex.axis=1.2, cex.lab=1.2, cex.main=2)
abline(a=0,b=1)
```

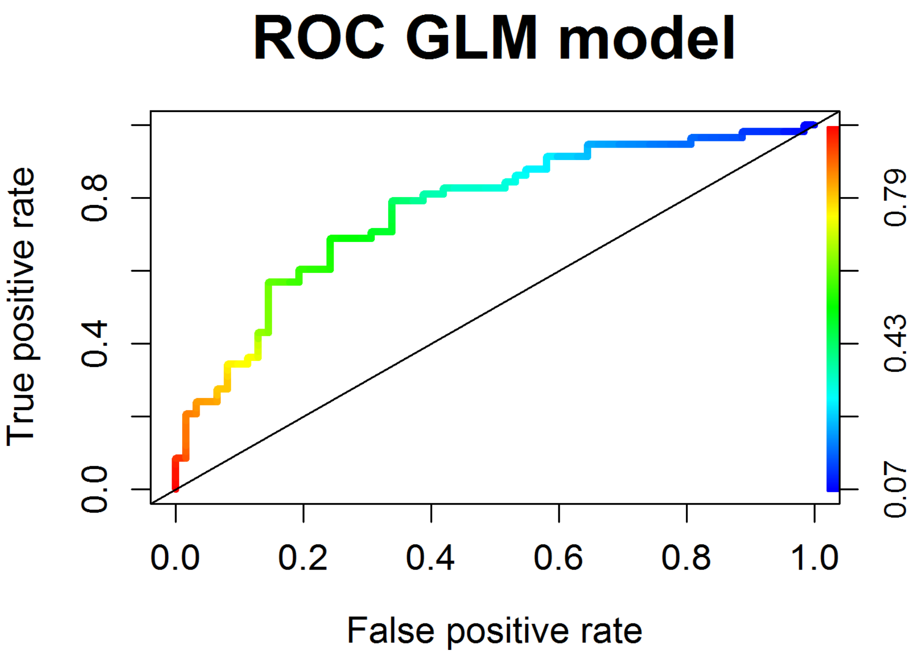


```r
# Cutoff can be chosen, higher cutoff favors sensibility, lower cutoff favors
specificity. Accuracy remains stable between 0.4 and 0.6.

plot(handeval, main="Accuracy GLM model", lwd=4, xaxis.cex.axis=1.2, yaxis.ce
x.axis=1.2, cex.lab=1.2, cex.main=2)
abline(v=0.528, h=0.73, col="red")
```

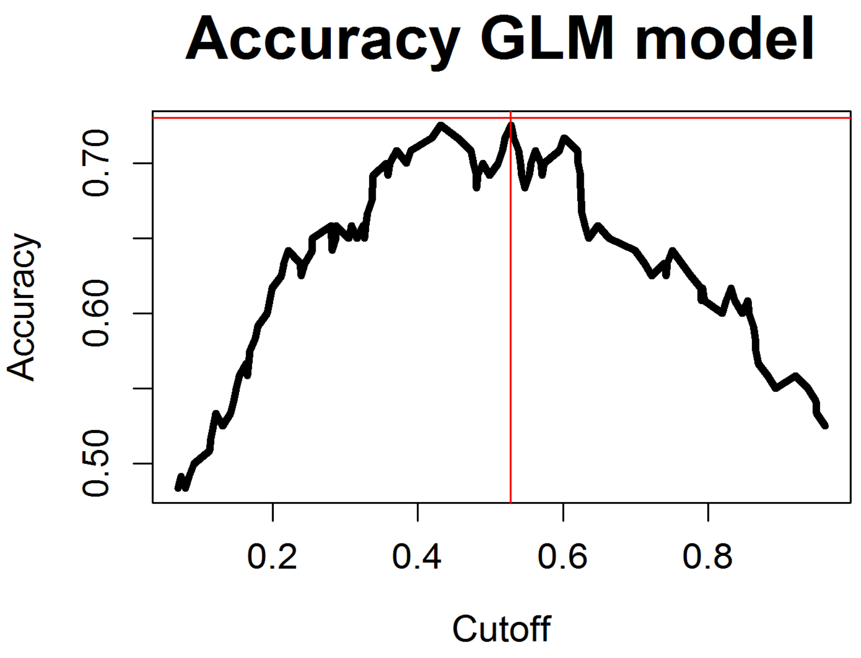

```r
plot(handsens, main="sensitivity GLM model", lwd=4, xaxis.cex.axis=1.2, yaxis
.cex.axis=1.2, cex.lab=1.2, cex.main=2)
abline(v=0.528, h=0.68, col="red")
```

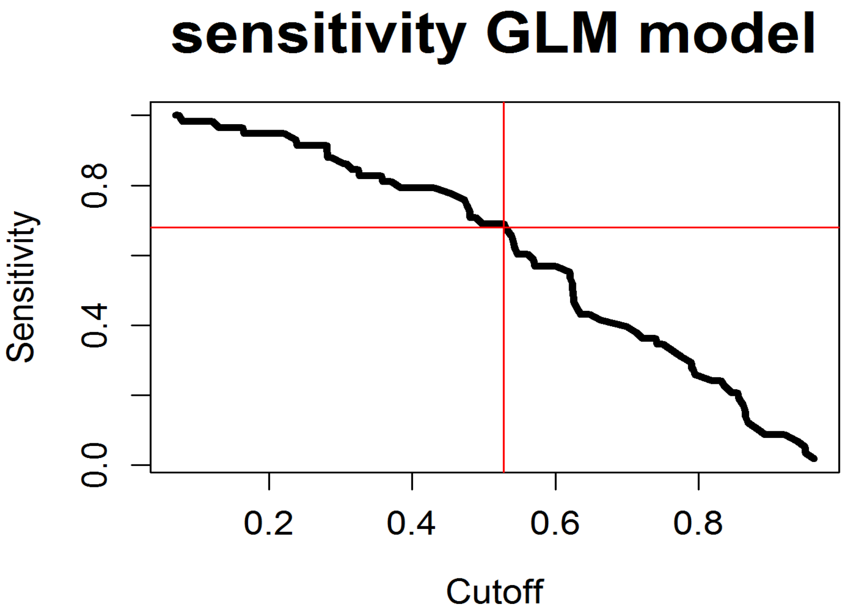


```r
plot(handspec, main="specificity GLM model", lwd=4, xaxis.cex.axis=1.2, yaxis
.cex.axis=1.2, cex.lab=1.2, cex.main=2)
abline(v=0.528, h=0.76, col="red")
```

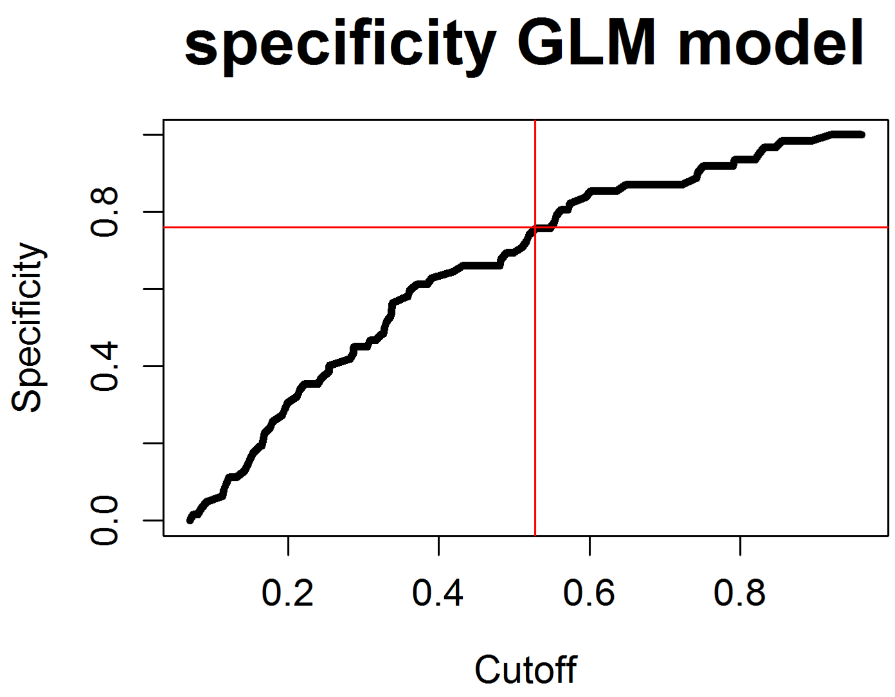


```
par(mfrow=c(1,1))

#plot predicted outcome to real outcome (1. make dataframe, sort data, rank d
ata high to low)
predictedvalues=data.frame(probability.of.ADA=outcomemean$x, ADA)
predictedvalues=predictedvalues[
  +order(predictedvalues$probability.of.ADA, decreasing=FALSE),]
predictedvalues$ranks = 1:nrow(predictedvalues)

ggplot(data=predictedvalues, aes(x=ranks, y=probability.of.ADA))+
  geom_point(aes(color=ADA), alpha=1, shape=4, stroke=2)+
  scale_colour_manual(name="ADA",values = c( "red", "blue"))+
  theme_minimal(base_size = 12)+
  geom_hline(yintercept = 0.528)
```

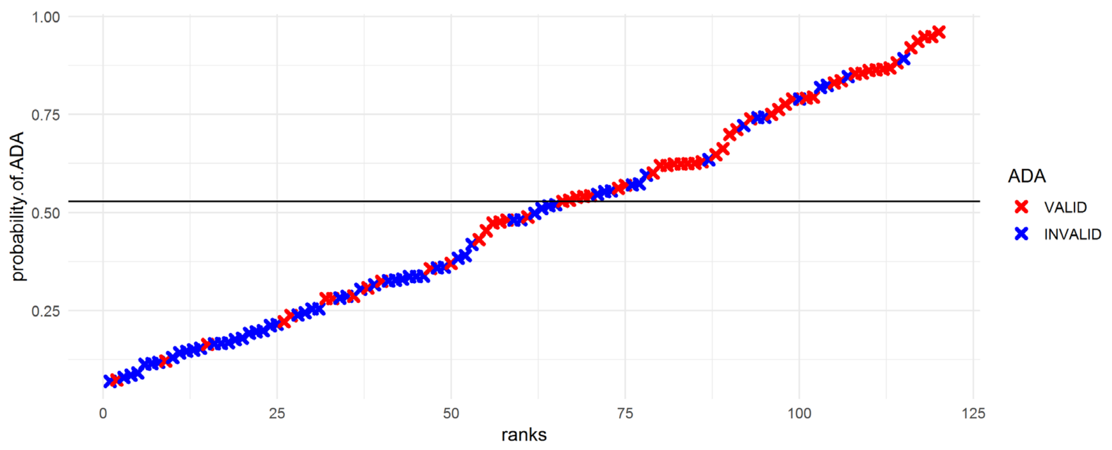


```
# RF model was built to test if the use of additional variables would increas
e predictive accuracy.
```

9. Random forest model (repeated CV)

```
Ab = factor(ifelse(imputedclean2$Ab == 1, "VALID", "INVALID"), levels = c("VA
LID", "INVALID"))

imputedclean2[, c("ADA", "random", "site", "dnalocsimpel", "ADATiter", "DNAmu
t")] = NULL

set.seed(42)
rf_model = train(y = Ab, x =imputedclean2, method = "rf", metric = "ROC",
                 trControl=trainControl(method="repeatedcv", number=10, repea
ts = 10, verboseIter = TRUE,
                                        classProbs = TRUE, savePredictions =
TRUE,
                                        summaryFunction = twoClassSummary))

print(rf_model)

## Random Forest
##
## 120 samples
##   6 predictor
##   2 classes: 'VALID', 'INVALID'
##
## No pre-processing
## Resampling: Cross-Validated (10 fold, repeated 10 times)
## Summary of sample sizes: 109, 108, 108, 108, 107, 108, ...
## Resampling results across tuning parameters:
##
##   mtry  ROC        Sens       Spec
##   2     0.7724603  0.7140000  0.6850000
##   4     0.7541627  0.7076667  0.6773810
##   6     0.7490556  0.7006667  0.6752381
##
```

```
## ROC was used to select the optimal model using the largest value.
## The final value used for the model was mtry = 2.
```

```
selectedmtry2 <- rf_model$pred [rf_model$pred$mtry== 2,]
predsRF= prediction(selectedmtry2$VALID, selectedmtry2$obs)
perfrf=performance(predsRF, "tpr", "fpr")
plot(perfrf, main="ROC RF model", colorize=TRUE, colorkey.relwidth=0.5,lwd=3,
     xaxis.cex.axis=1.2, yaxis.cex.axis=1.2, cex.lab=1.2, cex.main=2)
abline(a=0,b=1)
```

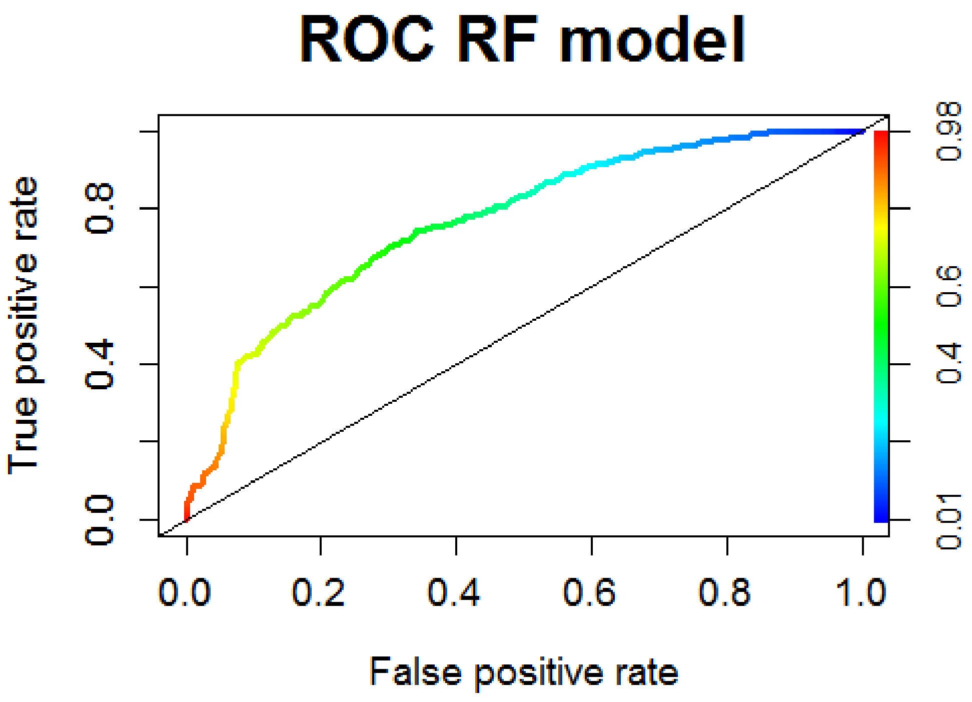

The random Forest model did not result in a better fit. Therefor data from the logistic regression model are represented in the article

10. Post hoc analyses (high risk location for missense mutation?)

```
# Although the model did not show a connection between mutation location and
iADA risk we hypothesized that within the missense group there might be some
hot-spot locations. (for example in AGALA epitopes or mutations that change c
onfiguration to hide epitopes)


missenselijst=imputedclean3[imputedclean3$mutatietype=='other',]
missenselijst$dnalocimputed=as.factor(missenselijst$dnalocimputed)
```

```r
# Missensemutations: DNA location is shown on the X-axis. Color represents iA
DA status. C.1025 seems to me prone for iADA development.
```

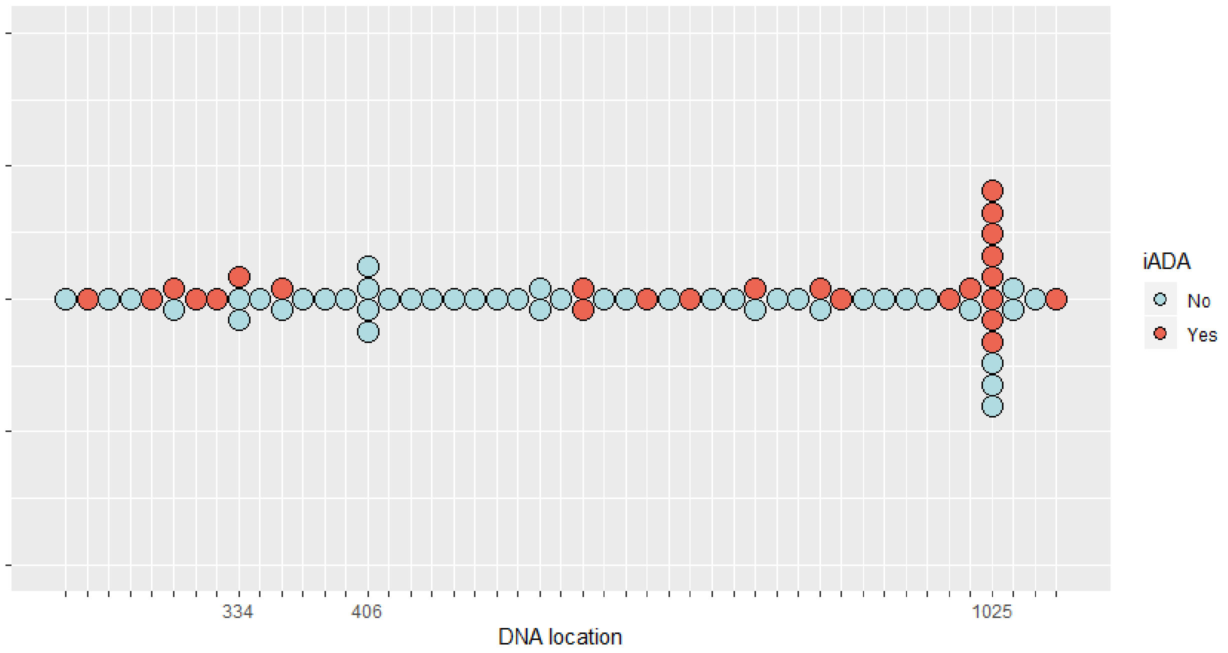


```r
# most prevalent mutationlocation is 1025 (all missense)
misenselijst$dnalocsimpel=ifelse(missenselijst$dnalocimputed==1025, "1025",
"Other")
missenselijst$dnalocsimpel=as.factor(missenselijst$dnalocsimpel)

Tabmismis=table(missenselijst$dnalocsimpel, missenselijst$Ab)
Tabmismis

##
##          0  1
##   Other 42 17
##   1025   3  8

fisher.test(Tabmismis)

##
##  Fisher's Exact Test for Count Data
##
## data:  Tabmismis
## p-value = 0.01279
## alternative hypothesis: true odds ratio is not equal to 1
## 95 percent confidence interval:
##   1.334264 41.856193
## sample estimates:
## odds ratio
##    6.38948
```

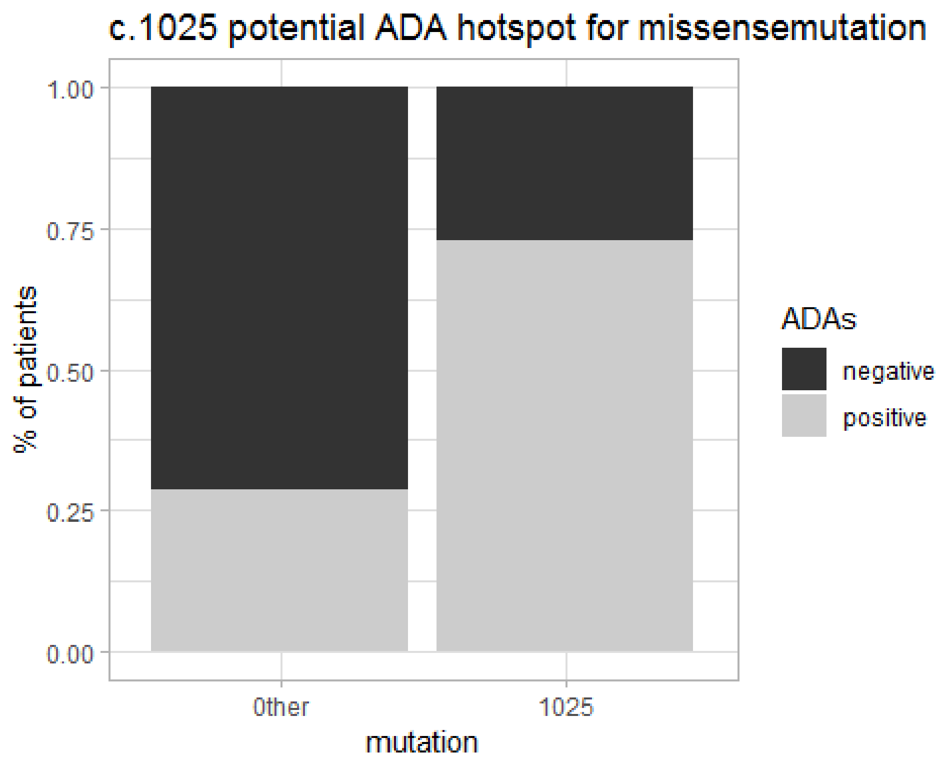

11. External cohort

```
# Due to different measuring techniques for iADA and lysoGb3, the second (ind
ependent) cohort could not be run in the initial model. To test reproducibili
ty, the same steps were repeated for the control cohort.

control<- read_excel([redacted]

control$`ADA`=as.factor(control$`ADA`)
control$`treatment`=as.factor(control$`treatment`)
control$`muttype`=as.factor(control$`muttype`)
control$muttype[control$muttype == "missense"] <- "other"

set.seed(42)
initialcontrol = mice(control, maxit=5)
predM = initialcontrol$predictorMatrix
predM[, c("Mutation")]=0
predM[, c("treatment")]=0
predM[, c("Initialdose")]=0
set.seed(42)
#Make 5 datasets
imputedcontrol=mice(control,predictorMatrix=predM, m=5)
plot(imputedcontrol)
```

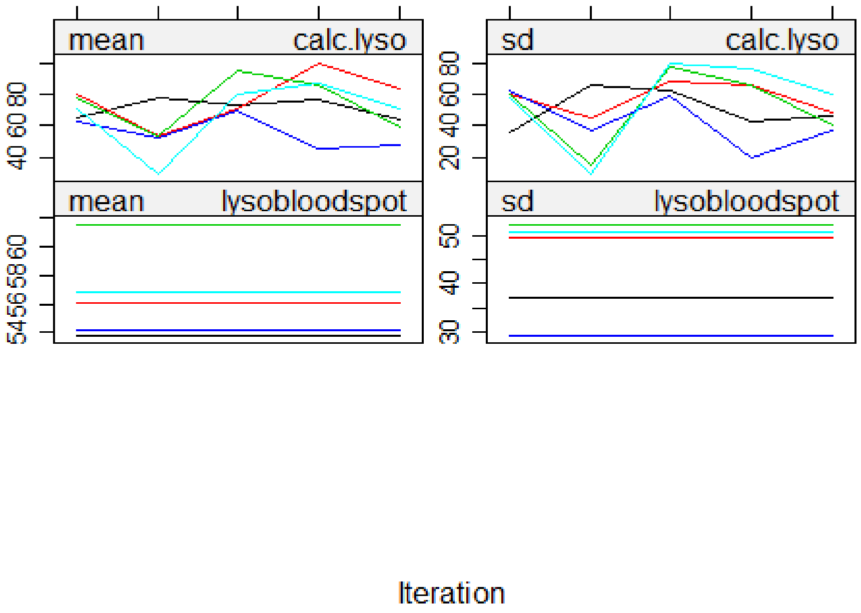


```
imputedcontrol <- mice::complete(imputedcontrol)

#calculated lysoGb3
```

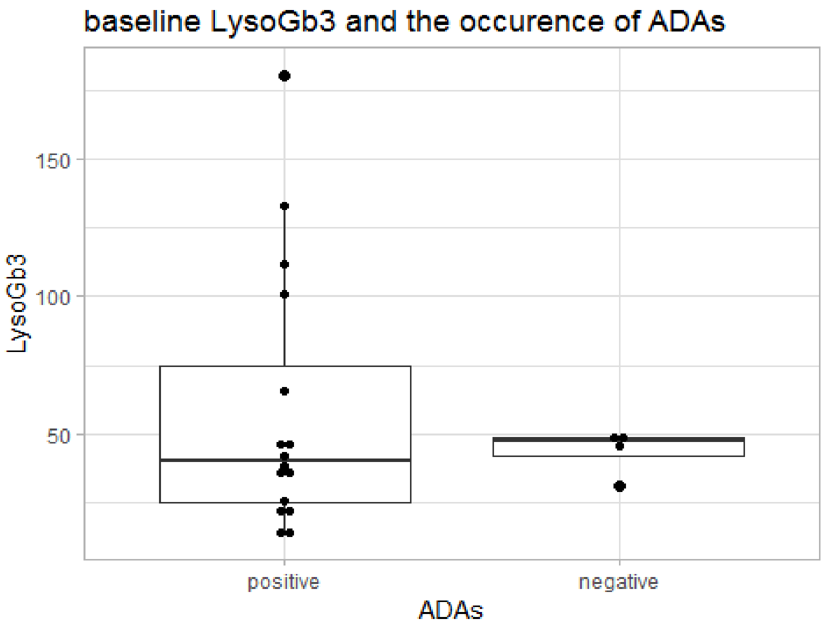


```
# mutation type
```

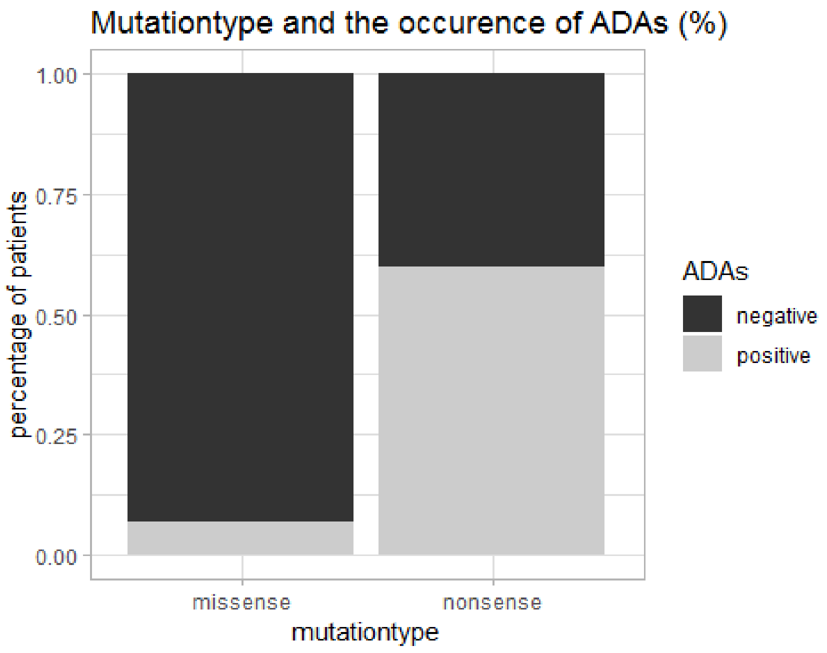


```
# treatment type
```

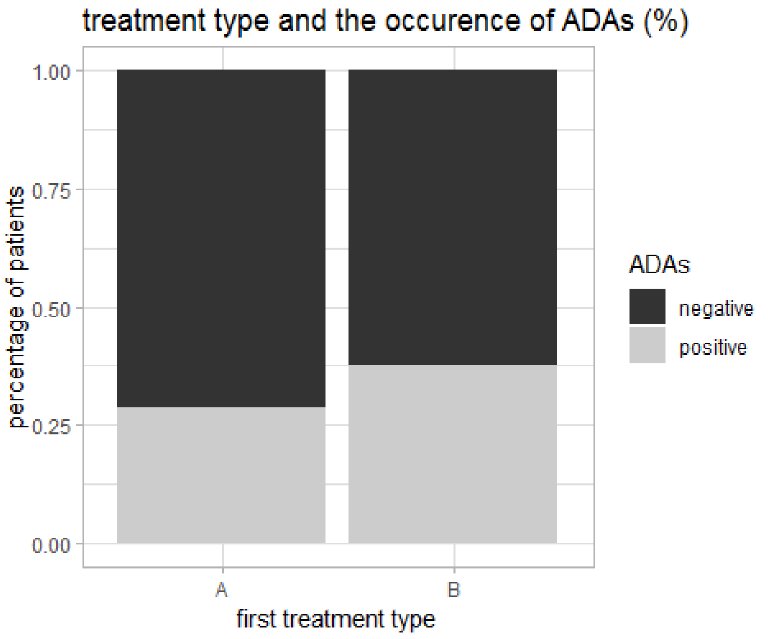


```
  # Repeated CV Logistic regression
ADA = factor(ifelse(imputedcontrol$ADA == 1, "VALID", "INVALID"), levels = c(
"VALID", "INVALID"))
imputedcontrol[, c("ADA","ADAtiter", "Initialdose", "Agestart", "Mutation", "
calc.lyso")] = NULL

set.seed(42)
```

```r
glm_control = train(y = ADA, x = imputedcontrol, method = "glm", metric = "RO
C",
                    trControl=trainControl(method="repeatedcv", number=10, repe
ats = 10,
                                           classProbs = TRUE, savePredictions =
TRUE,
                                           summaryFunction = twoClassSummary),
control = list(maxit = 50))
print(glm_control)
```

```
## Generalized Linear Model
##
## 30 samples
##  3 predictor
##  2 classes: 'VALID', 'INVALID'
##
## No pre-processing
## Resampling: Cross-Validated (10 fold, repeated 10 times)
## Summary of sample sizes: 27, 27, 27, 27, 27, 27, ...
## Resampling results:
##
##   ROC     Sens  Spec
##   0.8625  0.82  0.56
```

```r
varImp(glm_control)
```

```
## glm variable importance
##
##                Overall
## muttypeother    100.00
## treatmentB       10.31
## lysobloodspot     0.00
```

```r
summary(glm_control)
```

```
##
## Call:
## NULL
##
## Deviance Residuals:
##     Min       1Q   Median       3Q      Max
## -1.5300  -0.5515  -0.2933   0.7472   2.4921
##
## Coefficients:
##               Estimate Std. Error z value Pr(>|z|)
## (Intercept)   -4.36266    1.96938  -2.215   0.0267 *
## muttypeother   3.85843    1.55797   2.477   0.0133 *
## treatmentB     0.98991    1.05017   0.943   0.3459
## lysobloodspot  0.01224    0.01598   0.766   0.4435
## ---
## Signif. codes:  0 '***' 0.001 '**' 0.01 '*' 0.05 '.' 0.1 ' ' 1
```

```
##
## (Dispersion parameter for binomial family taken to be 1)
##
##     Null deviance: 38.191  on 29  degrees of freedom
## Residual deviance: 25.612  on 26  degrees of freedom
## AIC: 33.612
##
## Number of Fisher Scoring iterations: 5
```
